# Biosafety of mesoporous silica nanoparticles: a combined experimental and literature study

**DOI:** 10.1007/s10856-021-06582-y

**Published:** 2021-08-18

**Authors:** Lue Sun, Yu Sogo, Xiupeng Wang, Atsuo Ito

**Affiliations:** Health and Medical Research Institute, Department of Life Science and Biotechnology, National Institute of Advanced Industrial Science and Technology (AIST), Central 6, 1-1-1 Higashi, Tsukuba, Ibaraki 305-8566 Japan

## Abstract

Mesoporous silica (MS) particles have been explored for various healthcare applications, but universal data about their safety and/or toxicity are yet to be well-established for clinical purposes. Information about general toxicity of hollow MS (HMS) particles and about immunotoxicity of MS particles are significantly lacked. Therefore, acute toxicity and immunotoxicity of HMS particles were experimentally evaluated. A systematic and objective literature study was parallelly performed to analyze the published in vivo toxicity of MS particles. Lethal acute toxicity of MS particles is likely to arise from their physical action after intravenous and intraperitoneal administrations, and only rarely observed after subcutaneous administration. No clear relationship was identified between physicochemical properties of MS particles and lethality as well as maximum tolerated dose with some exceptions. At sub-lethal doses, MS particles tend to accumulate mainly in lung, liver, and spleen. The HMS particles showed lower inflammation-inducing ability than polyinosinic-polycytidylic acid and almost the same allergy-inducing ability as Alum. Finally, the universal lowest observed adverse effect levels were determined as 0.45, 0.81, and 4.1 mg/kg (human equivalent dose) for intravenous, intraperitoneal, and subcutaneous administration of MS particles, respectively. These results could be helpful for determining an appropriate MS particle dose in clinical study.

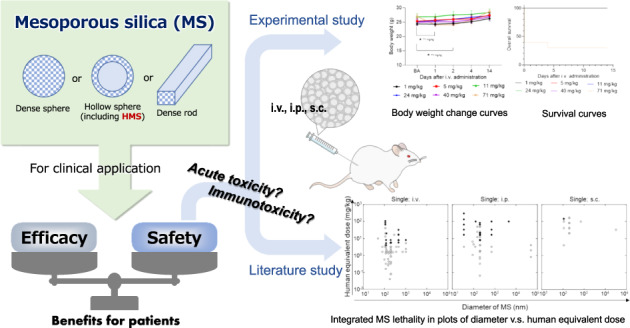

## Introduction

Universal data about the safety and/or toxicity of mesoporous silica (MS) particles are yet to be well-established in vivo. Especially, there is significant lack of information about general toxicity of hollow MS (HMS) particles, and about immunotoxicity of MS particles including HMS particles. MS particles efficiently retain drugs and biomolecules owing to their high surface areas, and controlled internal structure, pore size, pore structure, and surface functionality [1]. Thus, MS particles have been explored for various healthcare applications including drug delivery [2, 3], immunotherapy [4–6], tissue engineering [7], gene transfection [8], cell tracking [9], and food additive [10]. Before clinical applications, the toxicity level of MS particles needs to be established in order to ensure patient safety. However, only limited information about the toxicity of MS particles is available; even today, only limited information about the in vivo toxicity of MS particles is available from the viewpoint of clinical applications via i.p., i.v., and s.c. routes. Murugadoss et al. [11] and Napierska et al. [12], for examples, reviewed the toxicity of different types of silica nanoparticles with or without mesopores in various sizes. These research groups seemed to pay no attention to the presence/absence of mesopores on the silica nanoparticles in their reviews: they referred 13 papers in total for the cases of i.p.-, i.v.-, and s.c.-administration of silica nanoparticles, but in vivo toxicity of MS particles were discussed in only three papers among them. In that situation, it is unable to find the structure, size, or synthesis methods dependent toxicity of MS from their reviews. Thus, to the best of our knowledge, the in vivo toxicities of MS and HMS particles have not been comprehensively well-summarized yet.

On the other hand, host immunity plays crucial roles in anti-cancer therapies such as chemotherapy, radiotherapy, and immunotherapy in which MS particles attract interests. In chemotherapy, MS particles have been used as the carrier of chemotherapeutic drugs including doxorubicin, paclitaxel, and curcumin [13–15]. The drug-loaded MS particles demonstrated advantages of drug uptake in tumor cells in vitro and of anti-tumor effects in vivo as compared with the drugs alone. Meanwhile, curative efficacy of chemotherapy is linked to durable tumor-targeting immune responses [16, 17]. For example, a combination of the epifocal 2,4-dinitrochlorobenzene application with the systemic dacarbazine administration showed therapeutic effects on treatment of subcutaneous tumor in healthy C57BL/6 mice, but ineffective in immunodeficient RAG-1^−/−^ mice [18]. Systemic chemotherapy with doxorubicin and subcutaneous administration of B7-immunoglobulin G leads to cure of lymphoma in healthy C57BL/6 mice, but not to show any therapeutic effects in immunodeficient C57BL/6 severe combined immunodeficient mice [19]. On the contrary, adequate chemotherapy and radiotherapy can enhance the anti-tumor immune response through induction of immunogenic cell death [20]. In chemotherapy and immunotherapy, a series of MS particles with different size, pore size, hollow structure, etc., enhanced anti-tumor immune responses [4–6, 21–27]. The results show that MS and HMS particles are promising immunoadjuvants for cancer therapy owing to superior depot and immune-activating effects [26, 27]. About 20–30% of adsorbed tumor antigens were slowly released from the HMS particles over 1 week in vitro, indicating the HMS is a good carrier for cancer antigens [26]. Furthermore, administration of HMS particles loaded with tumor antigen prevented tumor growth in mice compared with administration of tumor antigens alone, or a mixture of tumor antigens and Alum that is a conventional immunoadjuvant [26].

As one of immunopotentiators, immunotoxicity of MS particles needs to be clarified in more detail. Immunopotentiators and immunoadjuvants, such as polyinosinic-polycytidylic acid (Poly(i:c)) [28], Freund’s adjuvant [29] and Alum [30] cause serious off-target effects. Poly(i:c) can induce arthralgia, fever, erythema, and sometimes life-threatening endotoxin-like shock [31]. Freund’s adjuvant can induce granulomas in the administration site, liver, and kidney [32]. Alum has allergy inducibility [33]. Thus, higher immune-activating potential and fewer off-target effects is an important focus when MS particles are applied to immune-linked therapies such as chemotherapy and radiotherapy of cancer, as well as immunoadjuvants for vaccines for cancer and infectious disease.

This study had three aims. The first aim was to perform experimental study to evaluate acute toxicity and immunotoxicity of HMS particles in mice in terms of the lowest lethal dose (LDLo), the maximum tolerated dose (MTD), the body weight change, cytokine, and IgE inducibility. The second aim was to analyze the published in vivo toxicity of MS particles through a systematic and objective literature study. The third aim was to acquire current whole aspects of toxicity of MS particles by the combination of the experimental and literature studies.

## Materials and methods

### Synthesis of HMS particles

HMS particles were synthesized by hydrolysis of tetraethyl orthosilicate in an ethanol aqueous solution containing hexadecyltrimethylammonium bromide and ammonia followed by structure transformation in hot ultrapure water, according to a previously published protocol [26]. The HMS particles have a size of 200 nm in diameter, a Brunauer–Emmett–Teller surface area of 1154 m^2^/g, a shell thickness of 30–40 nm, and mesopores of 3–6 nm [26].

### Preparation of HMS particle suspension for administration in mice

After dry sterilization at 180 °C, the HMS particles were aseptically suspended in physiological saline by ultrasonication using an UT-105S (SHARP, Osaka, Japan) for over 4 h.

### Single-dose toxicity testing as part of the safety evaluation of HMS particles

Male albino B6 mice were obtained from Charles River Laboratories Japan, Inc. (Tokyo, Japan). The mice were randomly grouped into s.c., i.p., and i.v. groups (*n* = 5–10), and administered with 250–2000, 23–260, or 1–71 mg/kg of HMS particles, respectively, as a single-dose. After the administration, the mice were monitored every day throughout the toxicological study. The body weights of the mice were measured on predetermined dates and compared with the body weights of those before administration to detect unintentional significant weight loss. The MTD was defined referring to previous papers [34, 35], as the maximum dose causing neither death nor serious adverse reactions (e.g., irregular breathing, over 10% weight loss and sluggish movement) in mice over 14 days post-administration.

### Cytokine and IgE inducibility of HMS particles in vivo

Cytokine and IgE inducibility of HMS particles were tested as part of the immunotoxicity evaluation. Cytokine levels in plasma were measured after HMS administration according to the previous report [31]. Briefly, 36 mg/kg of HMS, 36 mg/kg of Alum (LSL (Cosmo Bio), Tokyo, Japan), or 1.8 mg/kg of Poly(i:c) (FUJIFILM Wako Pure Chemical Corporation, Osaka, Japan) were s.c. or i.p administered to mice (*n* = 5). Blood was collected from the submandibular vein 1, 3, and 6 h after the administration. The plasma TNF, IL-6, and IL-10 levels were measured using the Cytometric Bead Array (Becton, Dickinson and Company, NJ, USA), according to the manufacturer’s protocol.

IgE titers in serum were measured according to the previous report [33]. Briefly, 36 mg/kg of HMS, 36 mg/kg of Alum, or 1.8 mg/kg of Poly(i:c) and endotoxin-free ovalbumin (OVA; FUJIFILM Wako Pure Chemical Corporation) were s.c. administered to mice at days 0, 14, and 21 for sensitization of mice with OVA (*n* = 5). The blood was collected from the submandibular vein at day 28. Total and OVA-specific IgE in plasma were measured using an ELISA kit (FUJIFILM Wako Shibayagi, Gunma, Japan), according to the manufacturer’s protocol.

### Literature study

**Fig. 1:**
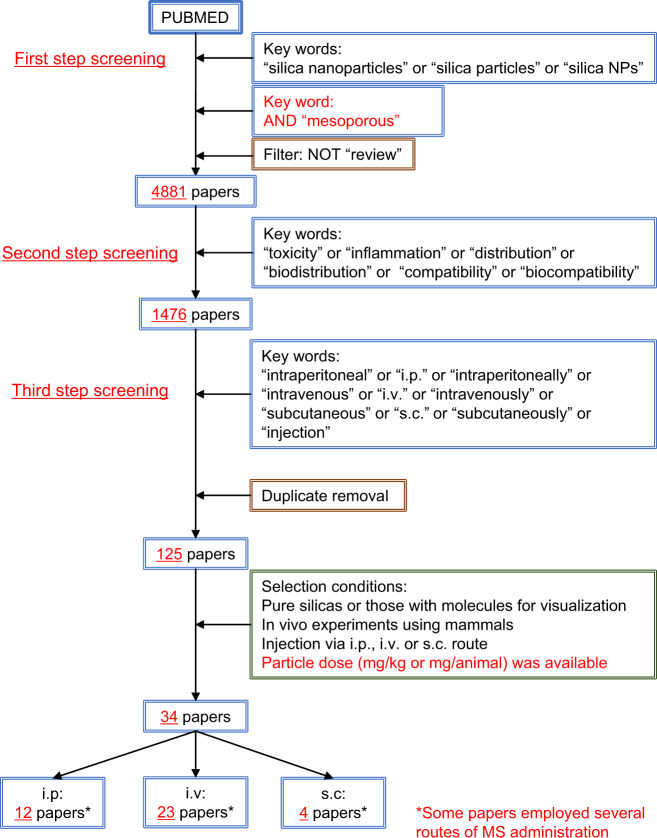
Process of literature search

The literature search was conducted on the PubMed^®^ database at the timepoint of June 18, 2020 (Fig. 1). The following keywords were applied to “All Fields” in combination: “silica nanoparticles” OR “silica particles” OR “silica NPs” AND “mesoporous” NOT “review” in the first-step screening. The retrieved literatures were screened for the following keywords applied to “All Fields”: “toxicity” OR “inflammation” OR “distribution” OR “biodistribution” OR “compatibility” OR “biocompatibility” in the second-step screening. Then the literatures were further screened for the following keywords applied to “All Fields”: “intraperitoneal” OR “i.p.” OR “intraperitoneally” OR “intravenous” OR “i.v.” OR “intravenously” OR “subcutaneous” OR “s.c.” OR “subcutaneously” OR “injection” in the third-step screening. Duplication of literatures was eliminated.

Then, literatures that satisfied all the following inclusion criteria were subjected to in-depth study: (1) MS being monolithic particles consisting of either pure silica, pure hydrated silica, or those plus labeling agents for pharmacokinetic evaluation, (2) MS examined in vivo in healthy or tumor-bearing animal model, (3) MS administrated by injection via i.p., i.v., and/or s.c. routes, (4) MS dose and follow-up period being disclosed, (5) administration schedule being disclosed in the case of repeated administration, and (6) diameter or length of MS particles being disclosed as their particle size. Note that the criterion (1) excludes literatures only on non-pure silica materials including metal-doped MS, mesoporous silicate, organic-modified MS, metal oxide coated with MS, and MS loaded with medicinal products or molecules. The doses of MS particles were converted to human equivalent doses (HED) in mg/kg [36].

### Statistical analysis

Numerical data for the experimental study were expressed in mean ± standard deviation. Student’s *t* test was used to analyze the statistical significance of differences. To detect unintentional significant weight loss, individual mice body weight data before and after administration of HMS particles were paired and analyzed. *p* < 0.05 was considered to indicate statistical significance.

## Results

### Acute toxicity of s.c. administration of HMS particles

**Fig. 2:**
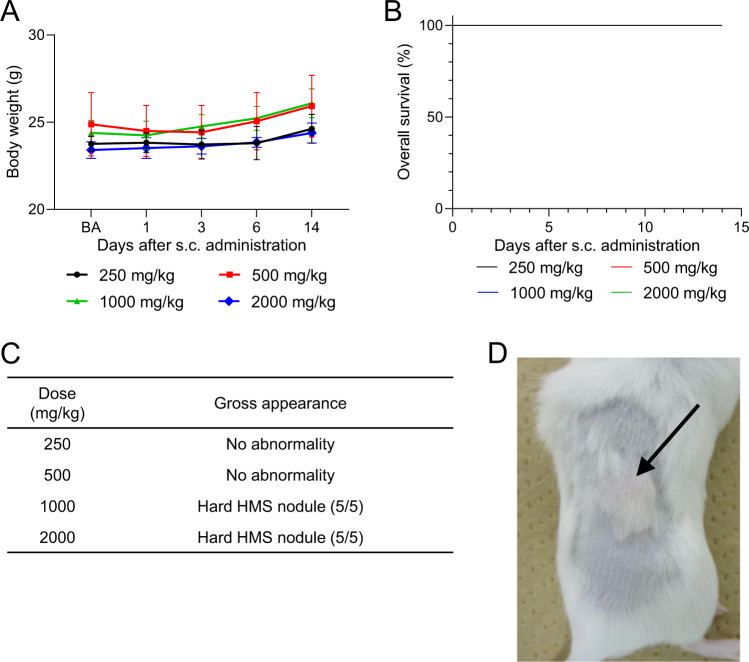
Observation results of the mice after subcutaneous administration with hollow mesoporous silica (HMS) particles. **A** Body weight changes. *p < 0.05 Student’s *t* test (vs. BA). **B** Overall survival. The survival curves for all the groups were overlaid because no mice were dead in these groups, and because the rates of overall survival maintained 100% throughout the single-dose toxicity test. **C** Summary of gross observation. **D** Photograph of hollow mesoporous silica nodule (arrow) 14 days after administration of 2000 mg/kg HMS particles. BA before administration of HMS particles

To evaluate the toxicity of s.c.-administered HMS particles, 250, 500, 1000, or 2000 mg/kg of HMS were administered by s.c. injection into mice. No significant body weight loss was observed in all groups (Fig. 2A). Neither death nor unusual behavior was observed in any groups (Fig. 2B, C). Nodule formation was observed at the administration site in the groups administered with high doses (1000 and 2000 mg/kg) of HMS particles (Fig. 2D). Daily activities of the mice were not limited with the nodules. These nodules became smaller with time and disappeared in ~2 months after administration (data not shown). Thus, the LDLo and MTD were estimated to be higher than 2000 mg/kg (163 mg/kg HED) for s.c. administration of the HMS particles.

### Acute toxicity of i.p. administration of HMS particles

**Fig. 3:**
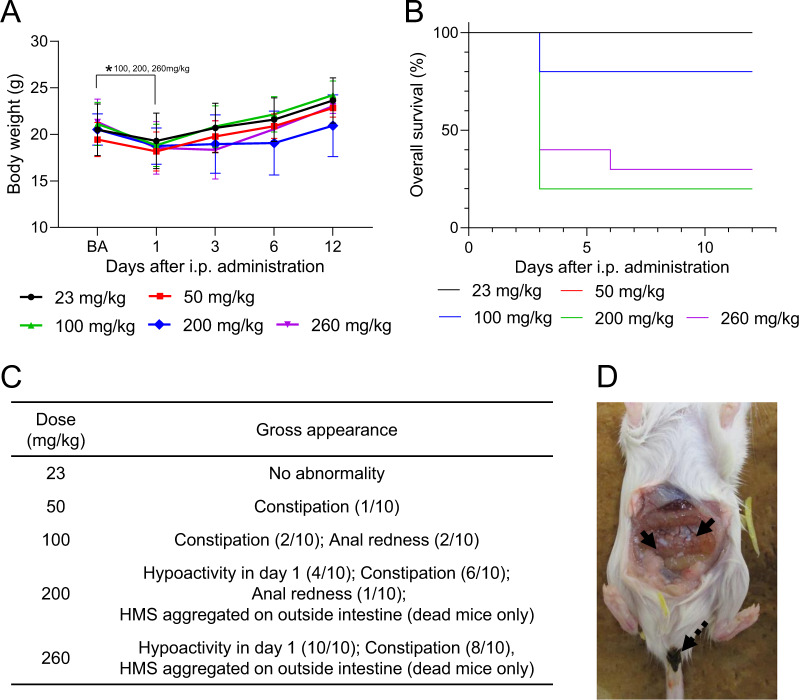
Observation results of the mice after intraperitoneal administration with HMS particles. **A** Body weight changes. *p < 0.05 Student’s *t* test (vs. BA). **B** Overall survival. The survival curves for the 23 and 50 mg/kg administration groups were overlaid because no mice were dead in both, and because the rates of overall survival maintained 100% throughout the single-dose toxicity test. **C** Summary of gross observation. **D** Photograph of hollow mesoporous silica aggregates (arrow) and constipation (dashed arrow) 3 days after administration of 260 mg/kg HMS particles. BA before administration of HMS particles

To evaluate the toxicity of i.p.-administered HMS particles, 23, 50, 100, 200, or 260 mg/kg of HMS particles were administered by i.p. injection into mice. The body weight significantly decreased by day 1 in the 100 mg/kg or higher administration groups (Fig. 3A). Part of mice died 3 days after administration at HMS doses of 100 mg/kg or higher (Fig. 3B). Constipation, anal redness, and hypoactivity were observed in all the administration groups except 23 mg/kg administration group (Fig. 3C). Necropsy of the dead mice revealed that HMS particles were aggregated on the outside of the intestine in the 200 and 260 mg/kg administration groups (Fig. 3D). The LDLo and MTD were estimated to be 100 mg/kg (8.1 mg/kg HED) and 50 mg/kg (4.1 mg/kg HED), respectively, for i.p. administration of HMS particles.

### Acute toxicity of i.v. administration of HMS particles

**Fig. 4:**
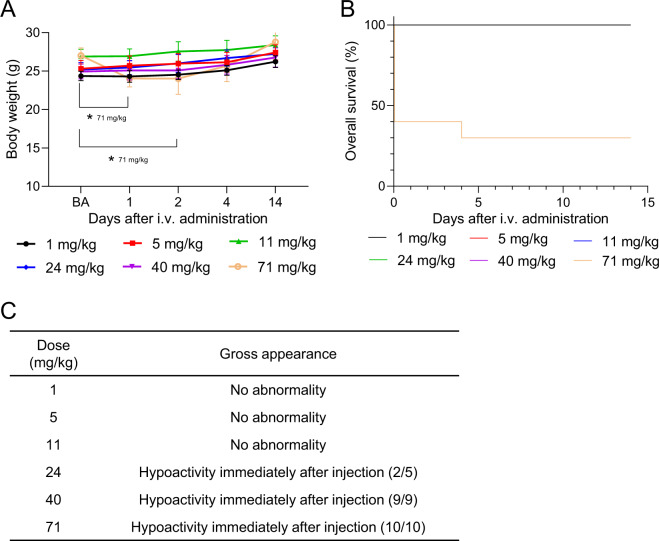
Observation results of the mice after intravenous administration with HMS particles. **A** Body weight changes. *p < 0.05 Student’s *t* test (vs. BA). **B** Overall survival. The survival curves for all except 71 mg/kg administration group were overlaid because no mice were dead in these groups, and because the rates of overall survival maintained 100% throughout the single-dose toxicity test. **C** Summary of gross observation. BA before administration of HMS particles

To evaluate the toxicity of i.v.-administered HMS particles, 1, 5, 11, 24, 40, or 71 mg/kg of HMS particles were administered by i.v. injection into mice. The body weight was significantly decreased in the 71 mg/kg administration group 1 day after injection (Fig. 4A). Half of mice died within 20 min after administration in the 71 mg/kg administration group while no mice died in other administration groups with doses of 40 mg/kg or less (Fig. 4B). Hypoactivity was observed immediately after administration of HMS particles at a dose of 24 mg/kg or more (Fig. 4C). The survived mice recovered from hypoactivity within 1 day after the HMS particles administration. Necropsy was performed for all the dead mice; no gross aberrations, such as bleeding in organs or aggregation of HMS particles, were observed. Thus, the LDLo and MTD were estimated to be 71 mg/kg (5.8 mg/kg HED) and 11 mg/kg (0.89 mg/kg HED), respectively, for i.v. administration of HMS particles.

### Cytokine inducibility of HMS particles

**Fig. 5:**
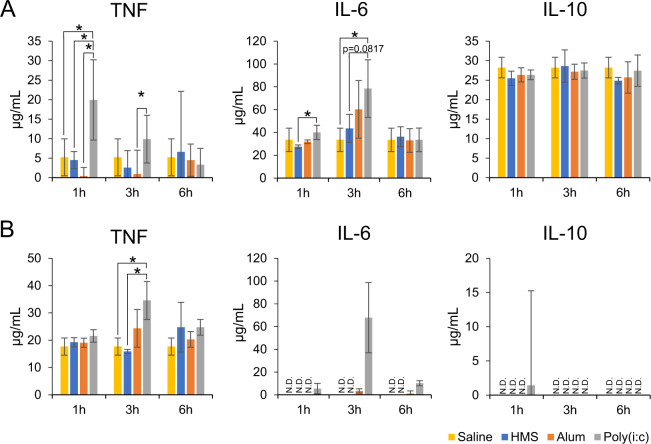
Cytokine induction of the mice administered with HMS particles, Alum, or polyinosinic-polycytidylic acid (Poly(i:c)). **A** Intraperitoneal administration. **B** Subcutaneous administration. *p < 0.05 Student’s *t* test. Saline administered group was as the negative control

The cytokine inducibility of HMS particles was compared with those of two well-known adjuvants, Alum and Poly(i:c). HMS particles and Alum unchanged cytokine levels after i.p. and s.c. administration (Fig. 5A, B). The HMS particle administration group showed significantly lower IL-6 levels than the Poly(i:c) administration group at 1 h after i.p. administration (Fig. 5A). The Poly(i:c) administration group also showed higher TNF and IL-6 levels than the HMS particle administration group after s.c. administration (Fig. 5B). These results showed that HMS particles was less toxic in vivo than Poly(i:c) and as toxic as Alum from the viewpoint of cytokine induction.

### IgE inducibility of HMS particles

**Fig. 6:**
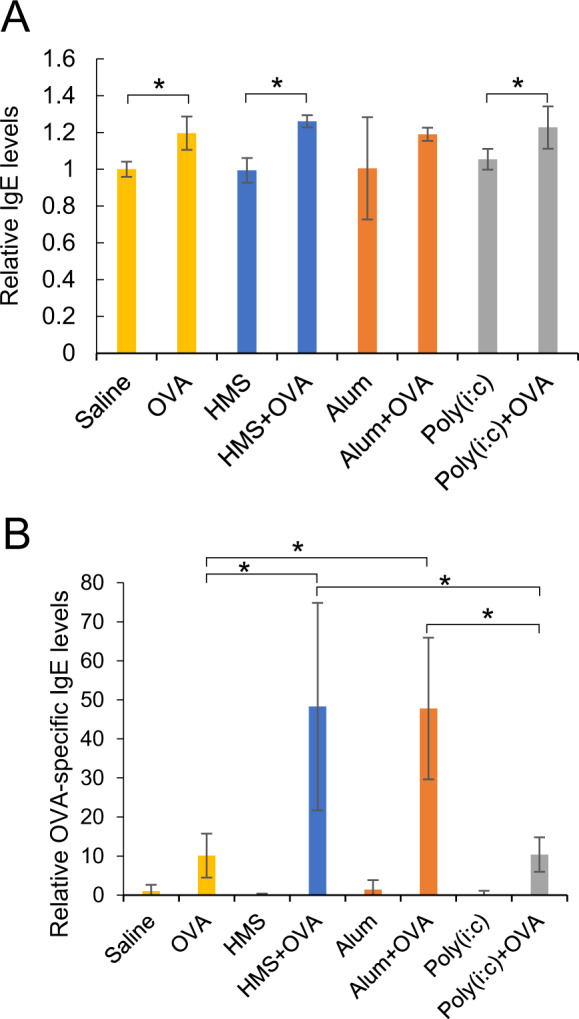
IgE induction in mice subcutaneously administered with HMS particles, Alum, or Poly(i:c). **A** Total IgE levels. **B** Ovalbumin (OVA) specific IgE levels. *p < 0.05 Student’s *t* test

Total and OVA-specific IgE levels were analyzed for mice sensitized with OVA in the presence or absence of HMS particles, Alum or Poly(i:c). Total IgE level in serum increased only when OVA was administered regardless of being administered with HMS particles, Alum or Poly(i:c) via s.c. or not. (Fig. 6A). These adjuvants alone unchanged total IgE levels (Fig. 6A). Administration of HMS particles or Alum in combination with OVA (HMS + OVA and Alum + OVA) via s.c. significantly increased the OVA-specific IgE level in serum compared with administration of OVA alone (Fig. 6B). The OVA-specific IgE levels were comparable between the HMS + OVA and Alum + OVA groups. These results showed that the toxicity level of HMS particles was the same as that of Alum from the viewpoint of total or antigen-specific IgE induction.

### Literature study of MS particles on in vivo toxicity

Thirty-four papers that reported in vivo responses to MS particles were retrieved after the keyword-based and content-based screening (Table S1). Ten of 34 papers adopt tumor-bearing animal models. Twenty-three, 12, and 4 papers report i.v., i.p., and s.c. administration studies, respectively. The earliest and latest dates of publication were October 2008 and May 2020, respectively. The diameters or long axes of MS particles ranged from ~50 to 50,000 nm.

#### Lethality (LDLo)

**Fig. 7:**
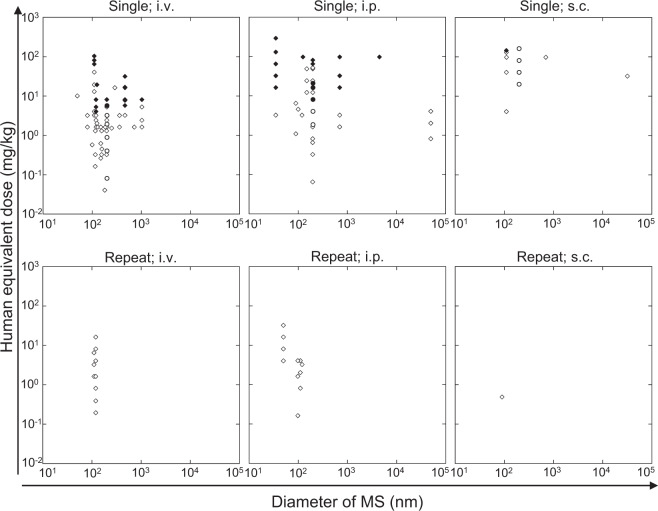
Integrated mesoporous silica lethality with different administration routes acquired by the combination of the experimental and literature studies. Open symbol: no animal death; filled symbol: at least one animal was dead; rhombus: data obtained in the literature study; circle: obtained in this study

Fifteen papers reported the lethality of MS particles. One of 15 papers employed a tumor-bearing and immunodeficient animal model. As shown in Fig. 7 and Table S1, the ranges of the reported LDLo for a single administration of MS particles were 4.1–81 mg/kg HED in i.v. administration, 16–65 mg/kg HED in i.p. administration, and 146 mg/kg HED in s.c. administration. No animal death was reported in repeated administration of 3.3–32 mg/kg HED of MS in i.v., 3.3–6.5 mg/kg HED of MS in i.p., and 0.49 mg/kg HED of MS in s.c. administrations (Fig. 7). Although the MS particles are tunable in size, shape (sphere or rod), internal structure (dense or hollow), the presence and absence of labeling agents, and process parameter (calcined or refluxed when surfactant was eliminated), no clear impacts of these factors were identified on lethality for both single and repeated administration except for the internal structure (Figs. 7 and S1–5S). LDLo of HMS particles (65–81 mg/kg HED) tended to be higher than that of dense MS particles (4.1–20 mg/kg HED) in i.v. administration (Fig. S4). Remarkably, the LDLo value of the i.v.-administered HMS particles was determined at 5.8 mg/kg HED in the present study (see “Cytokine inducibility of HMS particles”), and the value was comparable to the lower limit of the LDLo for the i.v.-administered MS particles obtained in the literature study.

#### MTD

Three papers reported MTDs of five types of MS particles in i.v. administration with one of three papers employing a tumor-bearing and immunodeficient animal model. MTDs for 500 nm MS particles were 7.7 and 3.3 mg/kg HED for female and male mice, respectively [34]. MTDs for 120, 190, and 1028 nm MS particles were 2.4, 2.4, and 5.3 mg/kg HED, respectively [35]. MTD for ~120 nm MS particles was 4.1 mg/kg HED [37]. One paper reported that MTD for 35 nm MS particles in i.p. administration was 3.3 mg/kg HED [38]. MTD increased by a 1.5–5-fold by modifying MS particles with primary amine groups [35].

#### Body weight

Fourteen papers reported body weights of animals after i.v. or i.p. administration of MS particles, among which 12 papers disclosed the concrete numerical data of body weights. Three of 14 papers employed tumor-bearing animal models [37, 39, 40]. Decreases in body weight within 3 days were reported in response to i.v. or i.p. single administration of 3.3–104 mg/kg HED or ≥12 mg/kg HED, respectively [34, 41, 42]. The i.v. single administration at 104 mg/kg HED was associated with suppressed weight-gain after 4 days [42]. Nine papers, including three tumor-bearing animal models, reported that the body weight gradually increased after single or repeated administration of MS particles dosed with 0.45–8.1 mg/kg HED [35, 37–40, 43–46] in i.v. or i.p. administration.

#### Biodistribution

Seventeen papers reported distribution of MS particles after i.v., i.p., and s.c. administration. Although five of 17 employed tumor-bearing animal models [40, 47–50], no clear differences in distribution were identified between the healthy and tumor-bearing animal models. MS particles were distributed in almost all organs after i.v. administration. MS particles were accumulated mostly in liver and spleen, fairly in lung, and slightly in other organs [34, 40, 42, 44, 48–56]. In liver, spleen and lung, the MS particles were accumulated within 5–30 min after i.v. administration [57, 58]. The accumulated MS particles remained longer in liver and spleen with a half-life of ~1 month [34, 40, 41, 57] while shorter in lung with a half-life of ~1–7 days [37, 57–59]. Distribution of MS particles after i.p. administration is reported in tumor-bearing animal models [13, 47]. The studies reported that MS particles were accumulated in liver, spleen, stomach, and intestine in 24 h after i.p. administration. These organs retained MS particles at least 1 week after administration [47]. S.c. administration of MS particles led to a slight increase in Si level in spleen 24 h after the administration, suggesting distribution of MS particles in spleen [52].

MS particles were excreted with the urine and feces [37, 44, 52, 57–59]. Interestingly, MS particles with the same appearance as those before administration were found in urine and feces at day 1 after administration [52, 59]. The results demonstrate that MS particles are excreted even without complete degradation for at least 1 day from administration.

#### Histology

Nineteen papers reported histological changes after administration of MS particles. After i.v. administration of 3.3–43 mg/kg HED of MS particles, histological aberrations were observed in liver [34, 35, 42], kidney [34, 35], lung [34, 35, 60], heart [34, 35], spleen [34, 60], and eye [34]. These papers showed that the typical aberrations caused by i.v. administration were embolism and inflammation-related damages, such as infarction and fibrosis. Notably, Mohammadpour et al. reported that histological aberrations in liver, lung, and spleen were apparently observed at day 10 after i.v. administration of ~3.3–7.7 mg/kg HED of MS particles 500 nm in diameter [34]. Increase in dose of MS particles in i.v. administration (~33–43 mg/kg HED) induced infarction-related abnormal lesions in lung, kidney, eye, and heart [34]. No aberrations were observed after i.v. administration of 0.45–4.1 mg/kg HED of MS particles [37, 39, 40, 44, 52, 57, 59, 61, 62].

After i.p. administration of 2.0–98 mg/kg HED of MS particles, histological aberrations were observed in liver [45, 46, 63], kidney [41], lung [60, 64], heart [64], and spleen [45]. These papers showed that the typical aberrations caused by i.p. administration were inflammation-related damages, such as fibrosis and increase in number of Kupffer cells in liver. However, in immunodeficient mice, no aberrations were observed in lung, kidney, liver, and intestine after i.p. administration of 4.1 mg/kg HED of MS particles [37].

After s.c. administration of 16 mg/kg HED of MS particles, red pulp expansion by foamy macrophages was observed in spleen [60]. After s.c. administration of 4.1 mg/kg HED of MS particles (110 nm in diameter), inflammation was observed at the administration site, while no aberrations were observed in spleen, lung, and liver [52]. After s.c. administration of 0.49 mg/kg HED of MS particles, no aberrations were observed [61].

#### Biochemical analysis

Thirteen papers reported biochemical analysis after i.v. or i.p. administration of MS particles. One of 13 papers employed a tumor-bearing and immunodeficient animal model [37]. Liver damage markers (ALT, AST, and ALP) after administration of MS particles are reported somewhat controversially. Five papers showed increases in the liver damage markers caused by i.p. administration of 0.81–49 mg/kg HED of MS particles, and by i.v. administration of 1.6–104 mg/kg HED of MS particles [37, 41, 42, 46, 65]. It should be noted that the lowest HED value of 0.81 mg/kg was recorded for extremely large MS particles (>50 µm) compared with other MS particles (110–198 nm). When this lowest value was excluded, i.p. administration of 4.1–49 mg/kg HED led to the increases in the liver damage markers. Dense and calcined MS tended to increase AST level in i.p. administration compared with refluxed MS [41, 65]. On the contrary, seven papers reported that no significant change was observed in the liver damage markers with doses ranging from 1.3 to 17 mg/kg HED after i.p. or i.v administration [34, 35, 43–45, 59, 66].

Kidney damage markers after administration of MS particles are also reported controversially. Two papers found increases in kidney damage marker (BUN) caused by i.p. administration of 24–49 mg/kg HED of MS particles, and by i.v. administration of 1.6 mg/kg HED of MS particles [41, 59]. However, eight papers reported that the kidney damage markers remained unchanged (2.0–104 mg/kg HED) [34, 35, 37, 42–46].

Thus, i.v or i.p. administration of MS particles may cause liver and kidney damages. The conjecture is consistent with the papers on biodistribution and histology after administration of MS particles. One paper evaluated cardiotoxicity, reporting increases in lactate dehydrogenase, total cholesterol and triglycerides after repeated i.p. administration of MS particles for 4 weeks (>4.0 mg/kg HED) [64].

#### Complete blood count

Eight papers reported changes in complete blood counts caused by MS particle administration. One of eight papers employed a tumor-bearing and immunodeficient animal model [37]. These papers reported contradictory results, being difficult to summarize them reasonably. For example, counts of white blood cells (or some of their main types like neutrophils, monocytes) increased in four papers [37, 42, 44, 64], while decreased in two papers [34, 59] after i.v. administration of MS particles. For another example, counts of platelets increased in two papers [34, 44] while decreased in one paper [64]. Two papers reported that blood counts remained unchanged after i.v. administration of MS particles [35, 43].

#### Cytokine inducibility

Collectively, MS particles are prone to activate inflammation pathway. Three papers reported increases in levels of inflammatory cytokines such as TNFα and IL-1β in serum 3–24 h after i.v. administration (0.45 mg/kg HED), and 4–8 week after i.p. administration (>2.0–4.0 mg/kg HED) of MS particles [40, 46, 64]. The increase after i.v. administration was that in tumor-bearing and immunodeficient mice.

Regarding cytokine levels in organs, one paper reported time-course changes in expression level of inflammatory and anti-inflammatory cytokines in liver, spleen, and lung after i.v. administration (3.3–7.7 mg/kg HED) of MS particles [34]. No increases were reported in TNFα and IL-6 levels in spleen 30 h after i.v. administration (1.3–1.7 mg/kg HED), and in IFN-γ level in splenocytes after s.c. administration (0.20 mg/kg HED) of MS particles [61, 66].

#### Antibody inducibility

One paper reported increases in IgG and IgM levels in serum after i.p. administration of MS particles (~98 nm; 20–50 mg/kg HED) [45].

#### Oxidative stress induction

Two papers reported oxidative stress induction after i.p. administration of MS particles. In heart and lung, reactive oxygen species and malondialdehyde were increased, while glutathione, catalase, superoxide dismutase, and glutathione peroxidase were decreased after repeated i.p. administration of MS particles (~50 nm; >4.0 mg/kg HED) [64]. In liver, 8-hydroxy-guanosine increased after repeated i.p. administration of MS particles (~50 nm; >2.0 mg/kg HED) [46].

#### Summary of MS dose that can induce adverse effects

MS dose ranges that can induce adverse effects were summarized for every routes of administration in Table 1. The MTD for i.v. administration and the LDLo for i.p. administration of the HMS particles in this experimental study were lower than those of MS particles reported in 34 papers. The literature study disclosed a fatal case of mice dosed 146 mg/kg HED MS particles via s.c. route although no mice were dead in our experiment even at 163 mg/kg HED HMS particles by s.c. administration. Taking the experimental and literature studies into account (Table 1), universal lowest observed adverse effect levels (uLOAEL) were found to be defined as the levels above which some adverse event occurs. The uLOAEL were estimated as 0.45, 0.81, and 4.1 mg/kg HED for i.v., i.p., and s.c. administration of MS particles, respectively.

**Table 1:** Summary of toxicity inducible dose of mesoporous silica particles in the unit of mg/kg HED

	Administration frequency	Lethality (Lowest published lethal dose)		Maximum tolerated dose		Body weight		Biodistribution	Histology	Biochemical analysis	Blood test	Cytokine	Antibody	Oxidative stress
		Experimental	Literature	Experimental	Literature	Experimental	Literature	Literature						
i.v.	Single	5.8	4.1–81	0.89	2.4–7.7	5.8	3.3–104	0.16–8.1 (1.6–8.1)^a^	3.3–43	1.6–41	1.6–41	0.45–7.7 (3.3–7.7)^a^	–	–
	Repeat	–	–	–	4.1	–	–	–	3.3	1.6–8.1	1.6	–	–	–
i.p.	Single	8.1	16–65	4.1	3.3	8.1	12	–	12–98	0.81–24	–	–	–	–
	Repeat	–	–	–	–	–	–	–	2.0–4.1	4.0–4.1	4.0–4.1	2.0–4.0	1.6	2.0–4.0
s.c.	Single	>163	146	>163	–	>163	–	4.1	4.1–16	–	–	–	–	–
	Repeat	–	–	–	–	–	–	–	–	–	–	–	–	–

^a^When the dose or dose range changed with limitation to discard the data for tumor-bearing mice, the changed ones were indicated in parentheses just below the original ones

## Discussion

Universal information about the safety and/or toxicity of MS particles was successfully obtained in this study. MS particles have been studied for various healthcare applications including drug carriers, cancer cell tracking tools, and immunoadjuvants for vaccines. Depending on the clinical applications, MS particles are assumed to be administered in the i.v., i.p., or s.c. route. The corresponding administration routes are, for example, i.v. for general drug carriers, i.p. for chemotherapy carriers for specific cancer like ovarian cancer, and s.c. for immunoadjuvants for vaccines. In general, safety and/or toxicity of a material depends on the administration routes. Previously, different MS particles were subjected to the safety and/or toxicity study in different administration routes depending on intended clinical purposes. Thus, the safety and/or toxicity data were applicable only to the specific MS particles in the specific administration route. To the best of our knowledge, there was no paper to show the universally available maximum safety dose of MS particles for i.v., i.p., and s.c. administrations. The present uLOAEL for the MS particles are the universal information determined by collecting dose data causing any kinds of adverse effects in various parameters (Table 1). The ranges of LDLo that indicate acute toxicity of the MS particles were estimated as 4.1–81, 16–65, and 146 mg/kg HED for i.v., i.p., and s.c. administrations, respectively. On the other hand, the uLOAEL were estimated as much lower values compared with the ranges of LDLo like 0.45, 0.81, and 4.1 mg/kg HED for i.v., i.p., and s.c. administrations of MS particles, respectively. The difference between the uLOAEL and LDLo suggests that various adverse effects caused by MS particle administration are unrelated to lethality. The uLOAEL could be useful to estimate the no observed adverse effect level that is described in the International Council for Harmonisation of Technical Requirements for Pharmaceuticals for Human Use guideline as important information for estimation of the first dose in humans.

Lethal acute toxicity of MS particles after i.v and i.p. administrations is likely to arise not from chemical but physical action of MS particles. In the mice death cases after administration of the HMS particles, most of the mice died within 20 min after i.v. administration while they were dead 3 days after i.p. administration (Figs. 3 and 4). The difference was probably caused not by chemical but by physical behavior of the HMS particles in vivo. The Si level in the mammalian body is significant [67], but whether Si has some essential function in vivo is not clear [68]. The toxicity of Si in animals has not been reported in the meaning of its chemistry. Thus, the acute toxicity is likely to be caused not by the immediate degradation of MS particles but by the physical action. It was reported that i.v.-administered MS particles immediately accumulated in liver, spleen, and lung within 30 min [57, 58]. I.v. administration of MS particles induced infarctions relating to abnormal lesion in organs, pulmonary embolism, and vasculature congestion [35, 36]. These results suggested that i.v. administration of MS particles has a risk to cause capillary embolism in a short period and subsequent immediate death. In mice death cases after i.p. administration of MS particles, some inflammatory lesions were also observed in lung [60, 64] or liver [46], but accumulation of MS particles in these organs tended to progress much slower (over 1 day [13, 47]) than the cases after i.v. administration. Thus, the cause of immediate death after i.p. administration could be somewhat different from that after i.v. administration. We found, after i.p. administration, HMS aggregated on the intestine and constipation (Fig. 3D). Similarly, MS aggregates were found on the intestine after i.p. administration; the authors similarly reported mortality of mice, but they mentioned no other signs or symptoms occurred on the mice [60]. These results suggested that formation of aggregates of MS in the intraperitoneal cavity is related to the death of mice. The occurrence of constipation in our experimental study suggests that HMS particles or their aggregates affect the intestine and defecation, but the mechanism and the relation to the death of mice is unclear.

Lethal acute toxicity was observed only rarely after s.c. administration of MS particles. Neither animal death nor serious adverse effects were observed after s.c. administration of HMS particles even at the dose of 163 mg/kg HED in our experimental study (Fig. 2). Although one paper reported animal death after s.c. administration of 146 mg/kg HED of MS particles [52], the dose was 2–35 times higher than that of i.v and i.p. administrations (Table 1). These results suggest that MS particles are administered more safely via s.c. route than via i.p. and i.v. routes. After s.c. administration of HMS particles, nodules were formed at the administration site, became smaller over time and finally disappeared a few months after the administration. This observation agreed with that of previous report on MS particles [69]. After s.c. administration of MS particles, Si level slightly increased in liver and spleen [52]. However, there is no histological evidence showing migration of MS particles in these organs [52]. These results suggested that s.c.-administered MS particles were gradually degraded and only degradation products were migrated to organs.

No clear relationship was identified between physicochemical properties of MS particles and lethality as well as MTD. Effects of size and shape of MS on lethality are somewhat controversial. Although some papers reported that smaller MS particles were more lethal than larger MS particles due to the higher risk of embolization [35, 58, 70], the literature study could not identify clear dependency of the lethality of MS particles on their shapes or the sizes. Regarding process parameters, surfactant molecules used for making mesoporous structure show toxicity in vitro [63, 71]. The surfactant molecules were removed by reflux with hydrochloric acid in 25 papers, or by calcination in ten papers including this study. It was suggested that methods of surfactant removal did not affect lethality of MS particles (Fig. S3). It should be noted that the reflux method is associated with difficulty to confirm complete elimination of surfactant from their MS particles. Additionally, MS particles modified by labeling agents such as fluorochrome or isotope showed no difference in lethality as compared with plain MS on i.v. administration (Figs. S1 and S2). Although only small number of data points are available in the literature study, it is suggested that sizes, shapes, preparation methods, and with/without modifications for labeling of MS particles do not largely, such as more than ten times, change toxicity of MS particles (Figs. S1–5S).

At sub-lethal doses, MS particles tend to accumulate mainly in lung, liver, and spleen, which can lead to organ damages. Typical damages in the organs with i.v. administration related to embolus and inflammation (thrombi, infarction, fibrosis). For i.p. administration, MS particles mainly induced inflammation-related damage, such as fibrosis and increase in Kupffer cells in liver. After s.c. administration, red pulp induced by foamy macrophages was observed in spleen. Tissue damage was observed after 3.3–98 mg/kg HED single MS particle administration and 2.0–4.1 mg/kg HED repeat administration (Table 1). However, in many cases, doses as low as 2 mg/kg HED of MS appear not to induce serious tissue damage. When the dose of MS particle increases, capillary embolization and saturation of phagocytosis by macrophages are likely to occur. Capillary embolization causes ischemia following local hypoxia (resulting in acute toxicity) and/or necrosis of tissue (chronic toxicity). Capillary embolization is probably accompanied with thrombosis, as some papers reported that platelets well adhere to silica particles to form aggregates potentially leading to platelet-mediated thrombosis. Saturation of phagocytosis by macrophages causes formation of silicotic lesions in tissues of the reticuloendothelial system [46, 63]. Although the formation of silicotic lesions is a rare event, it may be a typical toxic response to silica particles. However, there were no reports that the formation of silicotic lesions in organs caused death in the dosed animals. Thus, it is unlikely that the formation of silicotic lesions is directly related to the acute toxicity of MS particles, although it could relate to chronic toxicity, like necrosis of organs, as shown by some histological results.

Immunotoxicity of MS particles is at the same level as that of the immunoadjuvant, Alum, although MS particles induce inflammation-related cytokines. HMS particles were found to be inert for cytokines secretion after either i.p. or s.c. administration in mice although the cytokine levels of the mice significantly increased after Poly(i:c) administration via the same routes. One paper reported no increase in inflammation-related cytokines after MS particle administration [66] while others reported that MS particles have inflammation-related cytokines inducibility [34, 40, 46, 64]. We found that immunotoxicity of HMS is the same level as that of Alum from the viewpoint of cytokine induction and antigen-specific IgE induction, which agreed with the previous report [72]. These results suggested that MS particles induce no serious adverse effects relating to cytokines and allergy as compared with typical adjuvants, Poly(i:c) and Alum, used experimentally and clinically.

## Conclusion

In this experimental study, acute toxicity and immunotoxicity of HMS particles were evaluated. The LDLo and MTD for HMS particles were basically at the same levels as those of MS particles reported previously. HMS particles showed lower inflammation-inducing ability than Poly(i:c) and almost the same allergy-inducing ability as that of Alum. In the literature study, we summarized from various aspects the toxicity of MS particles administered in i.v., i.p., or s.c. routes. From the experimental and literature studies, it is concluded that the uLOAELs were determined as 0.45, 0.81, and 4.1 mg/kg HED for i.v., i.p., and s.c. administration of MS particles, respectively, regardless of size, shape (sphere or rod), internal structure (dense or hollow), the presence and absence of labeling agents, and the difference in elimination method of surfactant. These results could be helpful for determining an appropriate dose of MS particles, including the HMS, in clinical study.

## Supplementary Information

Supplementary Information
