# Supplementary material for: Biosafety of mesoporous silica nanoparticles: a combined experimental and literature study
Source: J Mater Sci Mater Med. 2021 Aug 18;32(9):102. doi: 10.1007/s10856-021-06582-y (PMC8373747; doi:10.1007/s10856-021-06582-y)
Supplement: Supplementary file 1 — Supplementary Information [file 10856_2021_6582_MOESM1_ESM.pdf]

# Supplementary Information

Article title: Biosafety of mesoporous silica nanoparticles: a combined experimental and literature study

Journal name: Journal of Materials Science: Materials in Medicine.

Author names: Lue Sun1)†, Yu Sogo 1)†, Xiupeng Wang 1) and Atsuo Ito1)

Affiliation: 1)Health Research Institute, Department of Life Science and Biotechnology, National Institute of Advanced Industrial Science and Technology (AIST), Central 6, 1-1-1 Higashi, Tsukuba, Ibaraki, 305-8566, Japan.

† Corresponding author; These authors contributed equally to this work.
Yu Sogo (Email: yu-sogou@aist.go.jp)
Lue Sung (Email: lue.sun@aist.go.jp)

Table S1. Summary of mesoporous silica *in vivo* safety obtained by the combined experimental and literature studies

| Particle | | Administration | | | | | Animal (Sex) | Results | | | | | | | | | | Components for imaging | References |
|---|---|---|---|---|---|---|---|---|---|---|---|---|---|---|---|---|---|---|---|
| Size | | Dose for an injection | | Route | Frequency | Follow-up period | | Lethality (Lowest published lethal dose) | Maximum tolerated dose | Body weight | Biodistribution | Histology | Biochemical analysis | Blood test | Cytokine | Antibody | Oxidative stress | | |
| TEM<br>nm | DLS<br>nm | Actual dose<br>mg/kg | Equivalent to human<br>mg/kg HED | | | | | | | | | | | | | | | | |
| 200 | - | 250-2000 | 20-163 | s.c. | Single | 14 days | Albino B6 mice (♂) | >163 mg/kg HED [a] | >163 mg/kg HED [a] | >163 mg/kg HED [a] | - | - | - | - | 2.9 mg/kg HED<br>TNF (-) (Serum)<br>IL-6 (-) (Serum)<br>IL-10 (-) (Serum) | 2.9 mg/kg HED<br>Total IgE (-) (Serum) | - | - | This study |
| 200 | - | 23-260 | 1.9-21 | i.p. | Single | 12 days | Albino B6 mice (♂) | 8.1 mg/kg HED | 4.1 mg/kg HED | ≥ 8.1 mg/kg HED : ↓* (day1) | - | - | - | - | 2.9 mg/kg HED<br>TNF (-) (Serum)<br>IL-6 (-) (Serum)<br>IL-10 (-) (Serum) | - | - | - | |
| 200 | - | 1-71 | 0.081-5.8 | i.v. | Single | 14 days | Albino B6 mice (♂) | 5.8 mg/kg HED | 0.89 mg/kg HED | 5.8 mg/kg HED : ↓* (day1) | - | - | - | - | - | - | - | - | |
| 466±86 | 799.8±22.9 | ~100 | ~8.1 | i.v. | Single | 1 year | BALB/c mice (♀ & ♂) | >8.1 mg/kg HED [a] | | not affected | - | - | ALT (-), AST (-), BUN (-), ALP (-), GLU (-) | NEU (-), EOS (-), BAS (-), LYM (-), MONO (-), WBC (-), RBC (-), MCV (-), HB (-), HCT (-), PLT (-), MCHC (-) | - | - | - | - | Mohammadpour et al. (2020) |
| ~50 (SEM) | N.A. | 25-200 | 4.0-32 | i.p. | Repeat (once a day, total 28 doses) | 4 weeks | Wistar rats (♂) | - | - | - | - | ≥4.0 mg/kg HED<br>Heart (+)<br>Lung (+) | ≥4.0 mg/kg HED<br>LDH↑*<br>T-COL↑*<br>TG↑* | ≥4.0 mg/kg HED<br>WBC↑*<br>32 mg/kg HED<br>RBC↓*<br>HB↓*<br>HCT↓*<br>PLT↓*<br>MCHC (-), MCV (-) | ≥4.0 mg/kg HED<br>TNF-α↑* (Serum) | - | ≥4.0 mg/kg HED<br>ROS, NO, MDA↑* (Heart)<br>SOD, CAT, GPx, GSH↓* (Heart)<br>ROS, NO, MDA↑* (Lung)<br>SOD, GPx, GSH↓* (Lung)<br>≥8.1 mg/kg HED<br>CAT↓* (Lung) | - | Hozayen et al. (2019) |
| 466±86 | 799.8±22.9 | 40-389 | 3.3-32 | i.v. | Single | 180 days | BALB/c mice (♀ & ♂) | 5.8(♂), 17(♀) mg/kg HED | 3.3(♂), 7.7(♀) mg/kg HED | ≥3.3(♂), 7.7(♀) mg/kg HED: ↓ (day1-3) | Lung, Liver, Spleen<br>(vs control) | ~3.3(♂), ~7.7(♀) mg/kg HED<br>Liver (+) (day10, 60, 180)<br>Lung (+) (day10)<br>Spleen (+) (day10)<br>~33(♂), ~41(♀) mg/kg HED<br>Kidney (+) (day10)<br>~43 mg/kg HED<br>Eye (+) (day10)<br>Heart (+) (day10) | 17 mg/kg HED<br>ALT (-), AST (-), BUN (-), ALP (-), GLU (-) | ≥3.3-7.7 mg/kg HED<br>NEU↓ (day10, 60)<br>LYM↓ (day10, 60)<br>MONO↓ (day10, 60)<br>MCHC↑ (day10)<br>PLT↑ (day10, 60) | mRNA expression (3.3-7.7 mg/kg HED)<br>IL-1β↑ (day10; Liver)<br>TNF-α, IL-4, IL-5, IL-6, IL-10, IL-12, IFN-γ↑ (day60; Liver)<br>IL-10↓ (day180; Spleen) | - | - | - | Mohammadpour et al. (2019) |
| 125±15 | 188±30 | 25 | 2.0 | i.v. | Single | 7 days | CD-1 mice (♀) | >2.0 mg/kg HED [a] | - | not affected | Liver, Spleen<br>(vs other organs) | Lung (-), Kidney (-), Liver (-), Spleen (-) | ALT (-), AST (-), BUN (-), ALP (-), GLU (-) | EOS ↑*<br>MONO ↑*<br>WBC ↑*<br>PLT ↑*<br>NEU (-), NEU (-), LYM (-), RBC (-), HB (-) | - | - | - | - | Moghaddam et al. (2019) |
| ~150 | N.A. | 7.7 | 0.63 | i.v. | Single | 54 hours | Balb/c mice (♀) | - | - | - | Liver, Spleen<br>(vs other organs) | - | - | - | - | - | - | $^{89}$Zr for PET imaging | Lee et al. (2019) |
| 115±15 | N.A. | 2 | 0.16 | i.v. | Single | 24 hours | Balb/c nude mice (♀) s.c. tumor bearing model | - | - | - | Lung, Liver<br>(vs control) | - | - | - | - | - | - | DiR for fluorescence imaging | Gao et al. (2019) |
| 100 | 163 | 3 | 0.58 | i.v. | Single | 8 days | Kun Ming mice (♀) s.c. tumor bearing model | - | - | not affected | - | Heart (-), Lung (-), Kidney (-), Liver (-), Spleen (-) | - | - | - | - | - | ZnPc for fluorescence imaging | Lin et al (2018) |
| N.A. | 158 | 5.56 | 0.45 | i.v. | Single | 21 days | Nude mice (N.A.) s.c. tumor bearing model | - | - | not affected | Liver, Spleen<br>(vs other organs) | Heart (-), Lung (-), Kidney (-), Liver (-), Spleen (-) | - | - | TNF-α↑ (3, 24 h; Serum)<br>IL-1β↑ (3, 24 h; Serum) | - | - | FITC for fluorescence imaging | Zhou et al (2018) |
| over 50000 (SEM, long axis) | N.A. | 10-50 | 0.81-4.1 | i.p. | Single | 7 days | Swiss albino mice (♂) | >4.1 mg/kg HED[a] | - | - | - | - | ≥0.81 mg/kg HED<br>ALT↑*<br>AST↑*<br>ALP↑* | - | - | - | - | - | Rawat et al (2017) |
| 190–390 (SEM) | N.A. | 200 | 16 | i.v. | Single | 72 hours | NMRI mice (N.A.) | - | - | - | Liver<br>(vs other organs) | - | - | - | - | - | - | $^{19}$F for MR imaging | Pochert et al (2017) |
| 180 | N.A. | 0.5 | 0.04 | i.v. | Single | 3 hours | SCID mice (♂) s.c. tumor bearing model | - | - | - | Lung, Liver, Spleen<br>(vs other organs) | - | - | - | - | - | - | $^{89}$Zr for PET imaging | Krammer et al (2017) |
| N.A. | 35 | 40-3600 | 3.3-293 | i.p. | Single | 10 days | BALB/c mice (♂) | 16 mg/kg HED | 3.3 mg/kg HED | 3.3 mg/kg HED: not affected | - | - | - | - | - | - | - | - | Mehravi et al. (2017) |
| 150 | N.A. | 20 | 1.6 | i.v. | Single | 24 hours | Athymic nude mice (♀) | - | - | - | Heart, Lung, Liver, Spleen, Stomach, Kidney, Ovary, Uterus, Intestine, Bladder<br>(vs control) | - | - | - | - | - | - | FITC and RhB for fluorescence imaging | Yang et al. (2016) |
| 115.8±16.4 | 238.5±7.7 | 4-16 | 0.33-1.3 | i.v. | Single | 30 hours | C57BL/6J mice (N.A.) | - | - | - | - | - | - | - | ≤1.3 mg/kg HED<br>TNF-α (-) (Spleen)<br>IL-6 (-) (Spleen) | - | - | - | Kwon et al. (2017) |
| 155.4±10.6 | 295.4±41.5 | 4-16 | 0.33-1.3 | i.v. | Single | 30 hours | C57BL/6J mice (N.A.) | - | - | - | - | - | - | - | 1.3 mg/kg HED<br>TNF-α (-) (Spleen)<br>IL-6 (-) (Spleen) | - | - | - | |
| 196.9±5.8 | 326.2±12.3 | 4-16 | 0.33-1.3 | i.v. | Single | 30 hours | C57BL/6J mice (N.A.) | - | - | - | - | - | 1.3 mg/kg HED<br>ALT (-), AST (-) | - | 1.3 mg/kg HED<br>TNF-α (-) (Spleen)<br>IL-6 (-) (Spleen) | - | - | - | |
| 196.9±5.8 | 326.2±12.3 | 0.8-20 | 0.065-1.6 | i.p. | Single | 24 hours | C57BL/6J mice (N.A.) | - | - | - | - | - | - | - | 1.6 mg/kg HED<br>TNF-α (-) (Serum)<br>TNF-α (-) (Peritoneal fluid) | - | - | - | |
| ~150 | N.A. | 150-600 | 12-449 | i.p. | Single | 12 days | BALB/c mice (♀) | - | - | - | - | ≥12 mg/kg HED<br>Liver (+) | - | - | - | - | - | - | Xi et al (2016) |
| 100 | N.A. | 56.2 | 4.6 | i.p. | Single | 24 hours | BALB/c nude mice (♂) | - | - | - | Liver, Spleen, Intestine<br>(vs other organs) | - | - | - | - | - | - | DiR for fluorescence imaging | Fu et al (2016) |
| N.A. | 250±76 | 18.8 | 1.5 | i.v. | Single | 48 hours | BALB/c mice (♀) | - | - | - | Liver, Spleen, Gastro-intestinal system<br>(vs other organs) | - | - | - | - | - | - | $Gd^{3+}$ for MR and $^{64}$Cu for PET imaging | Laprise-Pelletier et al. (2015) |
| 31900±1300 (long axis) | N.A. | 400 | 33 | s.c. | Single | 28 days | BALB/c mice (N.A.) | >33 mg/kg HED[a] | - | - | - | - | - | - | - | - | - | - | Choi et al (2018) |

Table S1. Summary of mesoporous silica *in vivo* safety obtained by the combined experimental and literature studies (continued)

| Particle | | Administration | | | | | Animal (Sex) | Results | | | | | | | | | | Components for imaging | References |
|---|---|---|---|---|---|---|---|---|---|---|---|---|---|---|---|---|---|---|---|
| Size | | Dose for an injection | | Route | Frequency | Follow-up period | | Lethality<br>(Lowest published lethal dose) | Maximum tolerated dose | Body weight | Biodistribution | Histology | Biochemical analysis | Blood test | Cytokine | Antibody | Oxidative stress | | |
| TEM<br>nm | DLS<br>nm | Actual dose<br>mg/kg | Equivalent to human<br>mg/kg HED | | | | | | | | | | | | | | | | |
| ~150 | N.A. | 3.2 | 0.26 | i.v. | Single | 21 days | BALB/c mice<br>(N.A.) | >0.26 mg/kg HED [a] | - | - | Liver, Spleen<br>(vs other organs) | - | - | - | - | - | - | $^{89}$Zr for PET imaging | Chen et al (2015) |
| N.A. | 198.2±21.8 | 150-1000 | 12-81 | i.p. | Single | 12 days | BALB/c mice<br>(♀) | 65 mg/kg HED | - | ≥12 mg/kg HED: ↓ (day1-3) | - | 24, 49 mg/kg HED<br>Kidney (+) (day2)<br>12-49 mg/kg HED<br>Kidney (+) (day12) | ≥24 mg/kg HED<br>AST ↑ * (day2)<br>BUN ↑ * (day2)<br>24 mg/kg HED<br>BUN ↑ * (day12)<br>49 mg/kg HED<br>ALT↑* (day2)<br>CRE↓* (day12) | - | - | - | - | - | Xi et al (2015) |
| 98±6 | N.A. | 2-50 | 0.16-4.1 | i.p. | Repeat<br>(5 days in a week,<br>total 20 doses) | 4 weeks | BALB/c mice<br>(♀) | >4.1 mg/kg HED [a] | - | 4.1 mg/kg HED: not affected | - | 4.1 mg/kg HED<br>Liver (+)<br>Spleen (+) | 4.1 mg/kg HED<br>ALT (-), AST (-), BUN (-), CRE (-) | - | - | ≥1.6 mg/kg HED<br>IgG ↑ (Serum)<br>IgM ↑ (Serum) | - | - | Lee et al (2013) |
| 80–100 | N.A. | 13.3-80 | 1.1-6.5 | i.p. | Single | 76 days | BALB/c nude mice<br>(♀)<br>i.p. tumor bearing model | - | - | - | Lung, Liver, Spleen, Intestine, Stomach<br>(vs other organs) | - | - | - | - | - | - | $^{165}$Ho for SPECT/CT imaging | Di Pasqua et al (2013) |
| 110 | 165 | 50-800 | 4.1-65 | i.v. | Single | 7 days | ICR mice<br>(♀) | 65 mg/kg HED | - | - | Lung, Liver, Spleen<br>(vs control) | 4.1 mg/kg HED<br>Lung (-), Kidney (-), Liver (-), Spleen (-) | - | - | - | - | - | FITC for fluorescence imaging | Fu et al (2013) |
| 110 | 165 | 50-1800 | 4.1-146 | s.c. | Single | 7 days | ICR mice<br>(♀) | 146 mg/kg HED | - | - | Liver, Spleen<br>(vs control) | 4.1 mg/kg HED<br>Injection site (+)<br>Lung (-), Kidney (-), Liver (-), Spleen (-) | - | - | - | - | - | FITC for fluorescence imaging | |
| 90±9 | N.A. | 6 | 0.49 | s.c. | Repeat<br>(every 2 week,<br>total 3 doses) | 6 weeks | C57BL/6J mice<br>(♀) | - | - | - | - | Heart (-), Lung (-), Liver (-), Spleen (-),<br>Kidney (-), Lymph nodes (-),<br>Injection site (-) | - | - | IFN-γ (-) | - | - | - | Mahony et al (2013) |
| 130 | N.A. | 20 | 1.6 | i.v. | Single | 48 hours | BALB/c mice<br>(♀) | - | - | - | - | Brain (-), Lung (-), Kidney (-), Liver (-),<br>Spleen (-), Testicle (-) | - | - | - | - | - | - | Wang et al (2013) |
| 120±25 | 268.9 | 20 | 1.6 | i.v. | Single | 72 hours | CD-1 mice<br>(♀) | - | - | - | Lung, Liver, Spleen<br>(vs control) | - | - | - | - | - | - | $^{125}$I for gamma counter measurement | Yu et al (2012) |
| 1028±139<br>(long axis) | N.A. | 20 | 1.6 | i.v. | Single | 72 hours | CD-1 mice<br>(♀) | - | - | - | Lung, Liver, Spleen<br>(vs control) | - | - | - | - | - | - | $^{125}$I for gamma counter measurement | |
| N.A. | 110 | 10-50 | 0.81-4.1 | i.p. | Repeat<br>(twice a week,<br>total 12 doses) | 8 weeks | ICR mice<br>(♀) | >4.1mg/kg HED [a] | - | ≤4.1 mg/kg HED: not affected | - | ≥2.0 mg/kg HED<br>Liver (+) | 4.1 mg/kg HED<br>ALT ↑ *<br>AST (-), BUN (-), CRE (-) | - | ≥2.0 mg/kg HED<br>TNF-α ↑ (Serum)<br>IL-1β ↑ (Serum) | - | ≥2.0 mg/kg HED<br>8-OHdG (Liver) | - | Liu et al (2012) |
| 120±25 | 208.6±1.5 | 30-100 | 2.4-8.1 | i.v. | Single | 10 days | CD-1 mice<br>(♀) | 4.1 mg/kg HED | 2.4 mg/kg HED | ≤8.1 mg/kg HED: not affected | - | 8.1 mg/kg HED<br>Heart (+)<br>Kidney (+)<br>Lung (-), Liver (-), Spleen (-) | 2.4 mg/kg HED<br>ALT (-), AST (-), BUN (-) | 2.4 mg/kg HED<br>WBC (-), RBC (-), HB (-), PLT (-) | - | - | - | - | Yu et al (2012) |
| 198±53<br>(long axis) | N.A. | 30-100 | 2.4-8.1 | i.v. | Single | 10 days | CD-1 mice<br>(♀) | 8.1 mg/kg HED | 2.4 mg/kg HED | ≤8.1 mg/kg HED: not affected | - | ≥5.3 mg/kg HED<br>Kidney (+)<br>8.1 mg/kg HED<br>Lung (+)<br>Liver (+)<br>Heart (-), Spleen (-) | 2.4 mg/kg HED<br>ALT (-), AST (-), BUN (-) | 2.4 mg/kg HED<br>WBC (-), RBC (-), HB (-), PLT (-) | - | - | - | - | |
| 1028±139<br>(long axis) | N.A. | 30-100 | 2.4-8.1 | i.v. | Single | 10 days | CD-1 mice<br>(♀) | 8.1 mg/kg HED | 5.3 mg/kg HED | ≤8.1 mg/kg HED: not affected | - | 8.1 mg/kg HED<br>Lung (+)<br>Kidney (+)<br>Heart (-), Liver (-), Spleen (-) | ≤5.3 mg/kg HED<br>ALT (-), AST (-), BUN (-) | ≤5.3 mg/kg HED<br>WBC (-), RBC (-), HB (-), PLT (-) | - | - | - | - | |
| 50 | N.A. | 124 | 10 | i.v. | Single | 24 hours | BALB/c nude mice<br>(♀)<br>s.c. tumor bearing model | - | - | - | Liver, Spleen<br>(vs other organs) | - | - | - | - | - | - | ZnPc for fluorescence imaging | Tu et al (2012) |
| 80 | 80 | 20 | 1.6 | i.v. | Single | 1 month | ICR mice<br>(♂) | >1.6mg/kg HED [a] | - | - | Lung, Liver, Spleen<br>(vs other organs) | Heart (-), Lung (-), Kidney (-), Liver (-),<br>Spleen (-) | - | - | - | - | - | FITC for fluorescence imaging | He et al (2011) |
| 120 | 120 | 20 | 1.6 | i.v. | Single | 1 month | ICR mice<br>(♂) | >1.6mg/kg HED [a] | - | - | Lung, Liver, Spleen<br>(vs other organs) | Heart (-), Lung (-), Kidney (-), Liver (-),<br>Spleen (-) | - | - | - | - | - | FITC for fluorescence imaging | |
| 200 | 200 | 20 | 1.6 | i.v. | Single | 1 month | ICR mice<br>(♂) | >1.6mg/kg HED [a] | - | - | Lung, Liver, Spleen<br>(vs other organs) | Heart (-), Lung (-), Kidney (-), Liver (-)<br>Spleen (-) | - | - | - | - | - | FITC for fluorescence imaging | |
| 360 | 360 | 20 | 1.6 | i.v. | Single | 1 month | ICR mice<br>(♂) | >1.6mg/kg HED [a] | - | - | Lung, Liver, Spleen<br>(vs other organs) | Heart (-), Lung (-), Kidney (-), Liver (-),<br>Spleen (-) | - | - | - | - | - | FITC for fluorescence imaging | |
| 80 | 80 | 20 | 3.2 | i.v. | Single | 8 hours | SD rats<br>(♂) | - | - | - | - | - | - | - | - | - | - | - | |
| 120 | 120 | 20 | 3.2 | i.v. | Single | 8 hours | SD rats<br>(♂) | - | - | - | - | - | - | - | - | - | - | - | |
| 200 | 200 | 20 | 3.2 | i.v. | Single | 8 hours | SD rats<br>(♂) | - | - | - | - | - | - | - | - | - | - | - | |
| 360 | 360 | 20 | 3.2 | i.v. | Single | 8 hours | SD rats<br>(♂) | - | - | - | - | - | - | - | - | - | - | - | |
| 185±22<br>(long axis) | N.A. | 20 | 1.6 | i.v. | Single | 18 days | Mice<br>(N.A.) | - | - | - | Liver<br>(vs other organs) | Lung (-), Kidney (-), Liver (-), Spleen (-) | BUN ↑ * (day1, 18)<br>ALT (-), AST (-), CRE (-) | WBC ↓ *†(day18)<br>MCV ↓ *†(day18)<br>RBC (-), HB (-), HCT (-), PLT (-) | - | - | - | FITC for fluorescence imaging | Huang et al (2011) |
| 720±65<br>(long axis) | N.A. | 20 | 1.6 | i.v. | Single | 18 days | Mice<br>(N.A.) | - | - | - | Spleen<br>(vs other organs) | Lung (-), Kidney (-), Liver (-), Spleen (-) | ALT (-), AST (-), BUN (-), CRE (-) | WBC ↓ *†(day18)<br>RBC (-), MCV (-), HB (-), HCT (-),<br>PLT (-) | - | - | - | FITC for fluorescence imaging | |

Table S1. Summary of mesoporous silica *in vivo* safety obtained by the combined experimental and literature studies (continued)

| Particle | | Administration | | | | | Animal (Sex) | Results | | | | | | | | | | Components for imaging | References |
|---|---|---|---|---|---|---|---|---|---|---|---|---|---|---|---|---|---|---|---|
| Size | | Dose for an injection | | Route | Frequency | Follow-up period | | Lethality (Lowest published lethal dose) | Maximum tolerated dose | Body weight | Biodistribution | Histology | Biochemical analysis | Blood test | Cytokine | Antibody | Oxidative stress | | |
| TEM nm | DLS nm | Actual dose mg/kg | Equivalent to human mg/kg HED | | | | | | | | | | | | | | | | |
| 110 | 110 | 40-1280 | 3.3-104 | i.v. | Single | 4 weeks | ICR mice (♀ & ♂) | 81 mg/kg HED | - | 104 mg/kg HED: ↓ (until day3) | Liver, Spleen (vs other organs) | ≥41 mg/kg HED<br>Liver (+) (day15)<br>104 mg/kg HED<br>Lung (-), Kidney (-), Spleen (-) (day15) | ≥41 mg/kg HED<br>ALT ↑ * (day15)<br>104 mg/kg HED<br>AST ↑ * (day15)<br>BUN (-), CRE (-), ALP (-), LDH (-) (day15) | ≥41 mg/kg HED<br>WBC ↑ †(day15)<br>104 mg/kg HED<br>RBC (-), MCV (-), HB (-), HCT (-), PLT (-), MCHC (-) (day15) | - | - | - | - | Liu et al (2011) |
| 110 | 110 | 20-80 | 1.6-6.5 | i.v. | Repeat (once a day for 2 weeks, total 14 doses) | 45 days | ICR mice (♀ & ♂) | >6.5 mg/kg HED [a] | - | not affected | - | ≥3.3 mg/kg HED<br>Liver (+) (day14)<br>6.5 mg/kg HED<br>Lung (-), Kidney (-), Spleen (-) (day14) | 6.5 mg/kg HED<br>ALT ↑ * (day14)<br>AST ↑ * (day14)<br>BUN (-), CRE (-), LDH (-) (day14) | - | - | - | - | FITC for fluorescence imaging | |
| 110–130 | N.A. | 50 | 4.1 | i.p. | Repeat (twice per week, total 18 doses) | 68 days | BALB/c nude mice (♀) s.c. tumor bearing model | >4.1 mg/kg HED [a] | - | not affected | - | Lung (-), Kidney (-), Liver (-), Spleen (-) Intestine (-), Stomach (-) | ALT ↑<br>AST ↑ | HCT ↑ | - | - | - | FITC for fluorescence imaging | Lu et al (2010) |
| 110–130 | N.A. | 10-200 | 0.81-16 | i.v. | Repeat (once per day, total 10 doses) | 10 days | BALB/c nude mice (♀) s.c. tumor bearing model | >16 mg/kg HED [a] | 4.1 mg/kg HED | ≤4.1 mg/kg HED: not affected | - | - | ≥8.1 mg/kg HED<br>ALT ↑ *<br>16 mg/kg HED<br>AST(-), BUN (-), CRE (-) | ≤4.1 mg/kg HED<br>WBC (-), MONO (-), LYM (-) | - | - | - | FITC for fluorescence imaging | |
| 110–130 | N.A. | 3-50 | 0.24-4.1 | i.v. | Repeat (twice per week, total 5 doses) | 14 days | BALB/c nude mice (♀) s.c. tumor bearing model | >4.1 mg/kg HED [a] | - | ≤3.3 mg/kg HED: not affected | - | 3.3 mg/kg HED<br>Heart (-), Lung (-), Kidney (-), Liver (-), Spleen (-), Muscle (-), Intestine (-), Great omentum (-), Stomach (-) | 1.6 mg/kg HED<br>ALT ↑ *<br>AST ↑ *<br>3.3 mg/kg HED<br>BUN (-), CRE (-), ALP (-), GLU (-), T-COL (-) | ≤1.6 mg/kg HED<br>NEU ↑ *<br>1.6 mg/kg HED<br>EOS ↑ *<br>3.3 mg/kg HED<br>WBC (-), RBC (-), MCV (-), HB (-), HCT (-), PLT (-), MCHC (-) | - | - | - | FITC for fluorescence imaging | |
| 110–130 | N.A. | 50 | 4.1 | i.v. | Single | 48 hours | BALB/c nude mice (♀) s.c. tumor bearing model | - | - | - | - | Lung (-), Kidney (-), Liver (-), Intestine (-) | - | - | - | - | - | FITC for fluorescence imaging | |
| 110–130 | N.A. | 50 | 4.1 | i.p. | Single | 96 hours | BALB/c nude mice (♀) s.c. tumor bearing model | - | - | - | - | - | - | - | - | - | - | FITC for fluorescence imaging | |
| 100–150 | 270±38 | 1200 | 98 | i.p. | Single | 40 hours | SV129 mice (♂) | ≤98 mg/kg HED [b] | - | - | - | - | - | - | - | - | - | - | Hudson et al (2008) |
| 100–150 | 470±252 | 1200 | 98 | i.p. | Single | 40 hours | SV129 mice (♂) | ≤98 mg/kg HED [b] | - | - | - | Lung (+) | - | - | - | - | - | - | |
| 600–800 | 740±160 | 20-1200 | 1.6-98 | i.p. | Single | 40 hours | SV129 mice (♂) | 16 mg/kg HED | - | - | - | - | - | - | - | - | - | - | |
| 4000–5000 | 4700±1150 | 1200 | 98 | i.p. | Single | 40 hours | SV129 mice (♂) | ≤98 mg/kg HED [b] | - | - | - | - | - | - | - | - | - | - | |
| 100–150 | 470±252 | 240 | 20 | i.v. | Single | 40 hours | SV129 mice (♂) | ≤20 mg/kg HED [b] | - | - | - | Lung (+) | - | - | - | - | - | - | |
| 100–150 | 270±38 | 1200 | 98 | s.c. | Single | 40 hours | SV129 mice (♂) | >98 mg/kg HED [a] | - | - | - | - | - | - | - | - | - | - | |
| 100–150 | 470±252 | 1200 | 98 | s.c. | Single | 40 hours | SV129 mice (♂) | >98 mg/kg HED [a] | - | - | - | - | - | - | - | - | - | - | |
| 600–800 | 740±160 | 1200 | 98 | s.c. | Single | 40 hours | SV129 mice (♂) | >98 mg/kg HED [a] | - | - | - | - | - | - | - | - | - | - | |
| 100–150 | 270±38 | 100 | 16 | s.c. | Single | 3 months | SD rats (♂) | - | - | - | - | Spleen (+) (day4) | - | - | - | - | - | - | |
| 100–150 | 470±252 | 100 | 16 | s.c. | Single | 3 months | SD rats (♂) | - | - | - | - | Spleen (+) (day4) | - | - | - | - | - | - | |
| 600–800 | 740±160 | 100 | 16 | s.c. | Single | 3 months | SD rats (♂) | - | - | - | - | - | - | - | - | - | - | - | |
| 4000–5000 | 4700±1150 | 100 | 16 | s.c. | Single | 3 months | SD rats (♂) | - | - | - | - | Spleen (+) (week2) | - | - | - | - | - | - | |

Route: s.c.: subcutaneous; i.p.: intraperitoneal; i.v.: intravenous

Animal: N.A.= not assigned

Lethality: a: no animals were dead at the maximum dose of MS during the experimental period; b: animals were dead during the experimental period, but they did not analyze lower dose

Maximum tolerated dose: a: no animals were dead at the maximum dose of MS during the experimental period

Body weight: *: statistically significant change; ↑ increase; ↓ decrease; a: no animals were dead at the maximum dose of MS during the experimental period

Histology: (+) found some kind of abnomal region; (-) did not find any abnomal region

Biochemical analysis: ALT: alanine aminotransferase; AST: aspartate aminotransferase; BUN: blood urea nitrogen; CRE: creatinine; ALP: alkaline phosphatase; GLU: glucose; LDH: lactate dehydrogenase; T-COL: total cholesterol; TG: triglycerides; *: statistically significant change; (-): no remakble change; ↑ increase; ↓ decrease

Blood test: WBC: white blood cells; NEU: neutrophils; EOS: eosinophils; LYM: lymphocytes; MONO: monocytes; BAS: basophils; RBC: red blood cells; HB: hemoglobin; HCT: hematocrit; PLT: platelet; *: statistically significant change; (-): no remakble change; ↑ increase; ↓ decrease; †: within normal renge

Cytokine: *: statistically significant change; (-): no remakble change (vs control); ↑ increase; ↓ decrease

Antibody: *: statistically significant change; (-): no remakble change (vs control); ↑ increase; ↓ decrease

Oxidative stress: *: statistically significant change; (-): no remakble change (vs control); ↑ increase; ↓ decrease

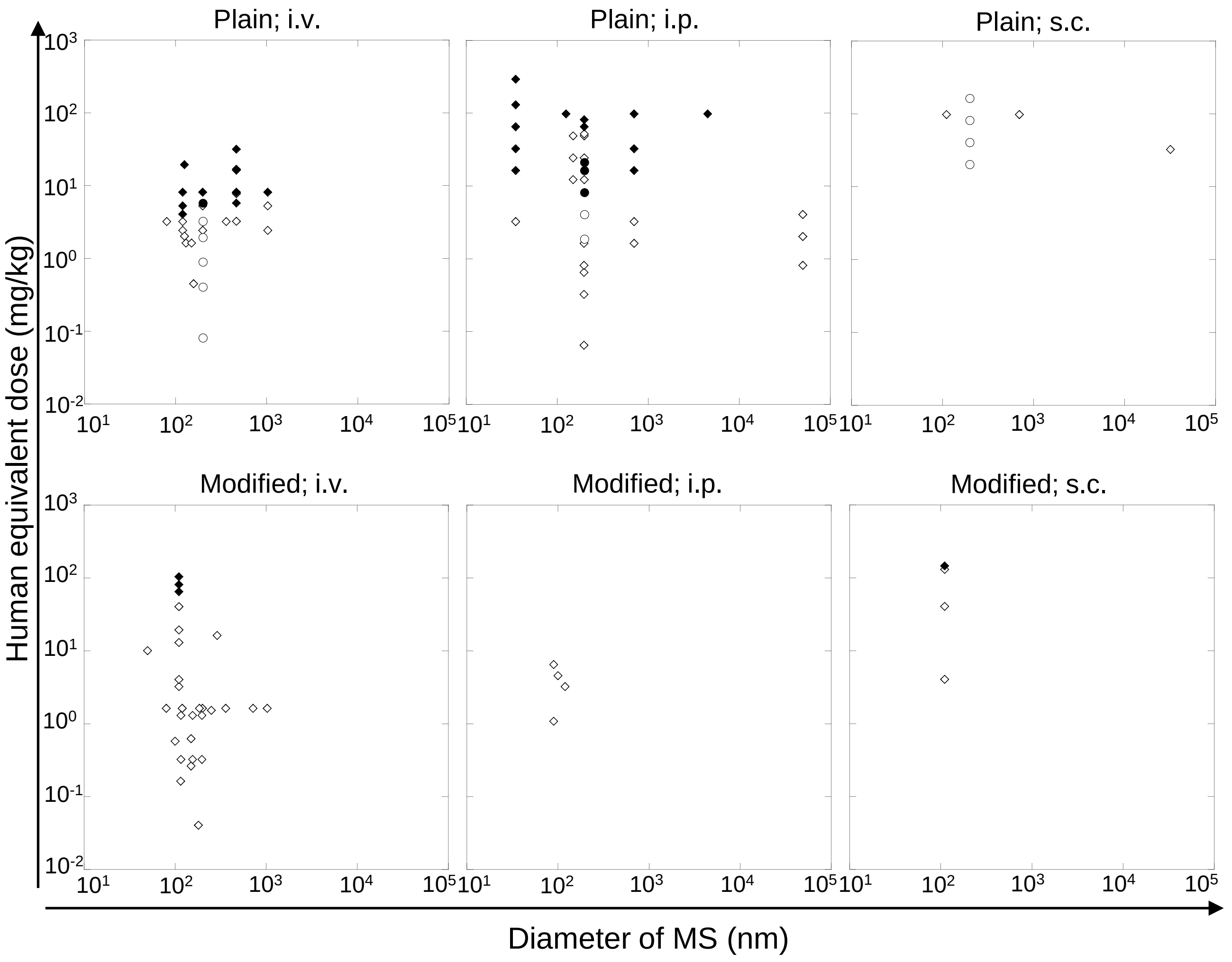


**Fig. S1 Single administration of plain MS particles or those modified by labeling agents**

Open symbol: no animal death; filled symbol: at least one animal was dead; rhombus: data obtained in the literature study; circle: obtained in the experimental study.

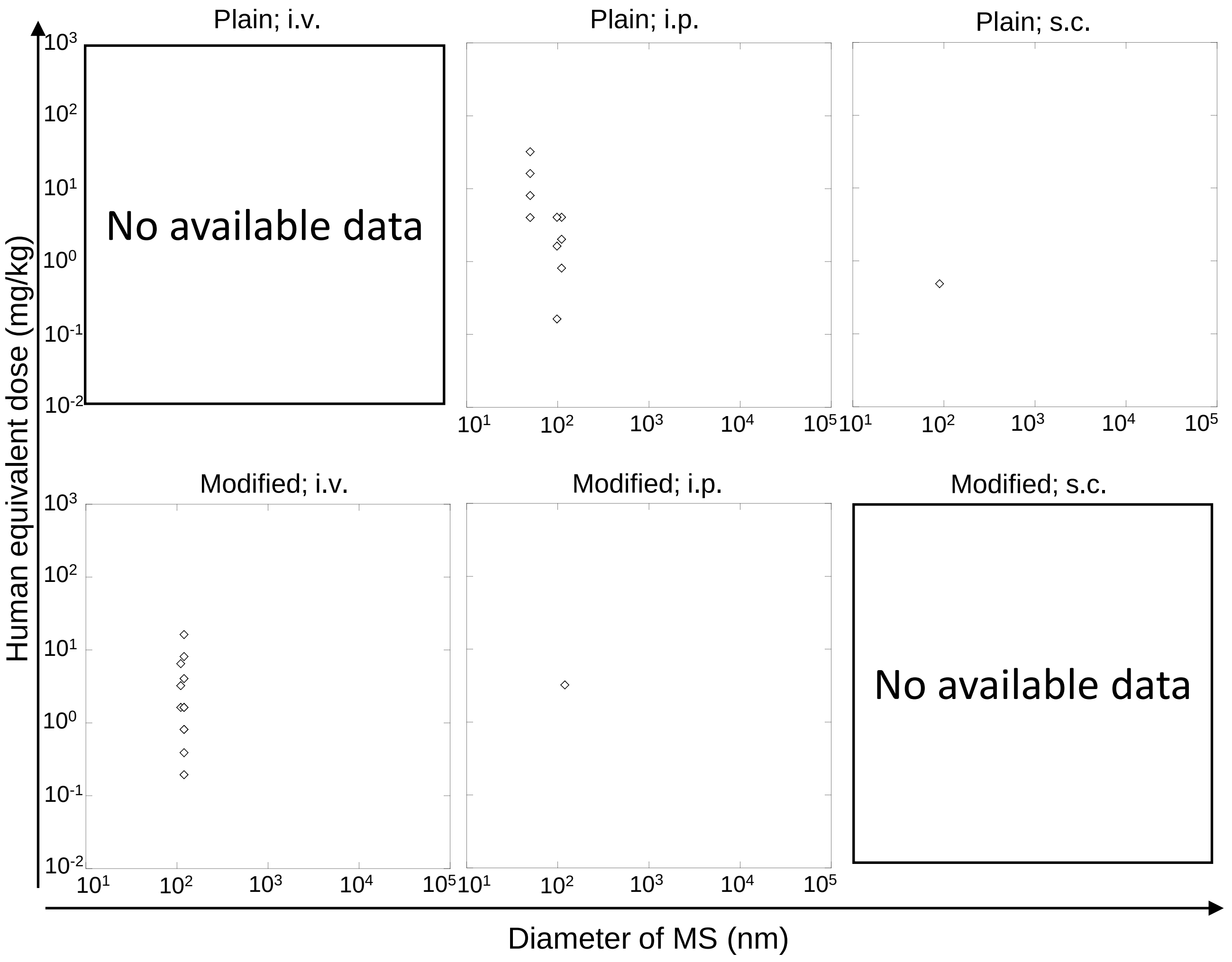


**Fig. S2 Repeated administration of plain MS particles or those modified by labeling agents**

Open symbol: no animal death; filled symbol: at least one animal was dead; rhombus: data obtained in the literature study; circle: obtained in the experimental study.

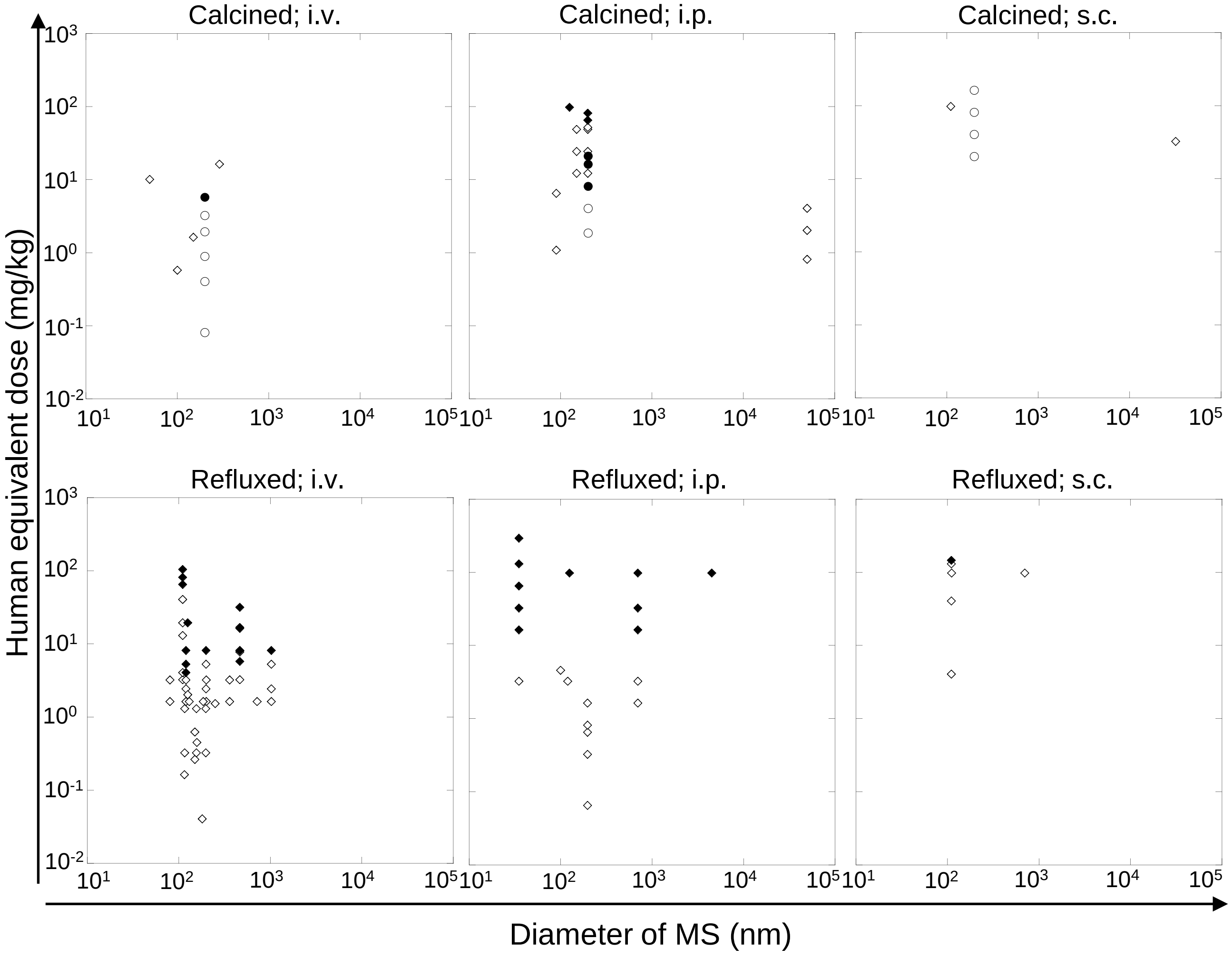


**Fig. S3 Single administration of calcined or refluxed MS particles for elimination of surfactant**

Open symbol: no animal death; filled symbol: at least one animal was dead; rhombus: data obtained in the literature study; circle: obtained in the experimental study.

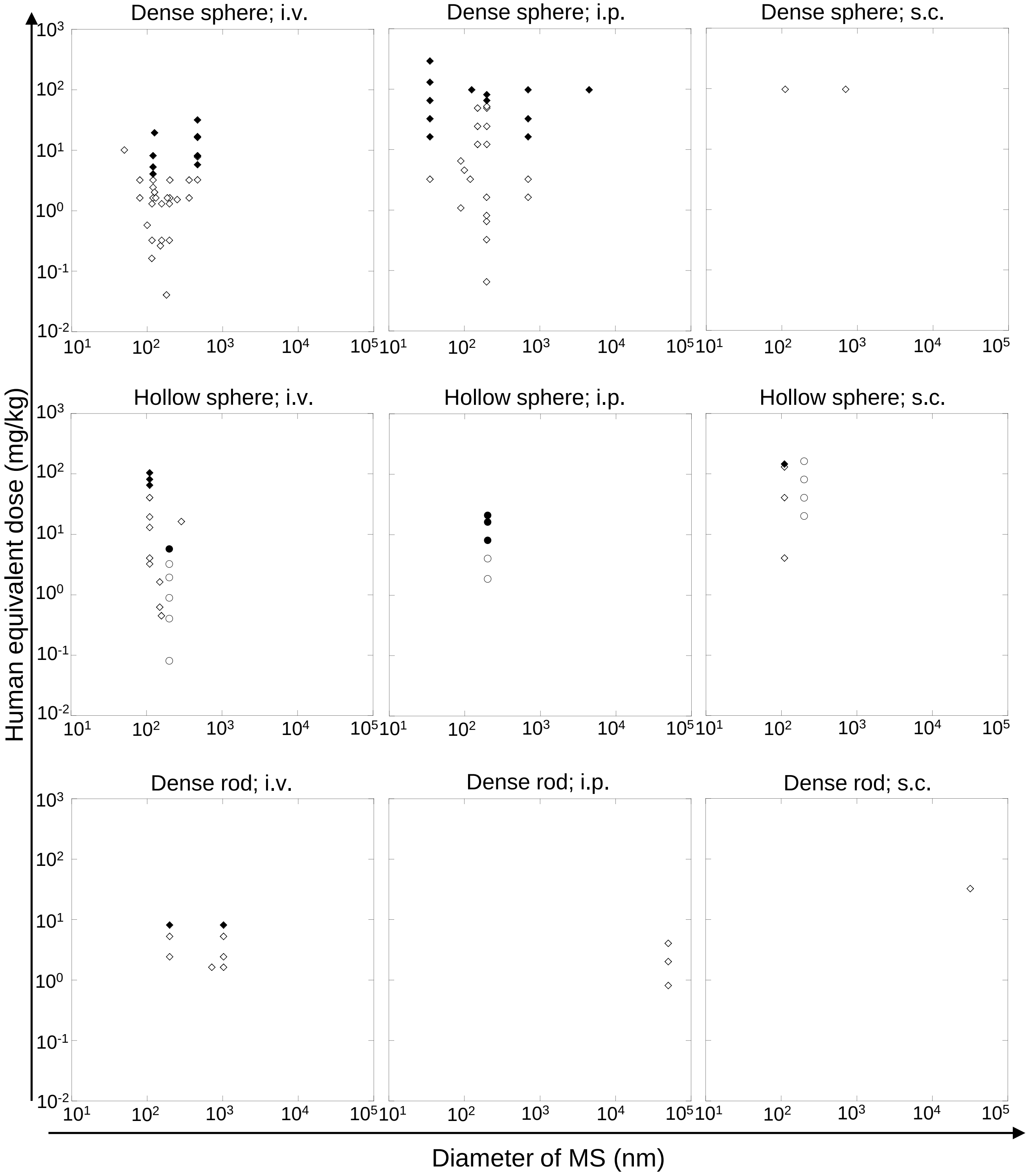


**Fig. S4 Single administration of MS particles with various shapes and internal structures**

Open symbol: no animal death; filled symbol: at least one animal was dead; rhombus: data obtained in the literature study; circle: obtained in the experimental study.

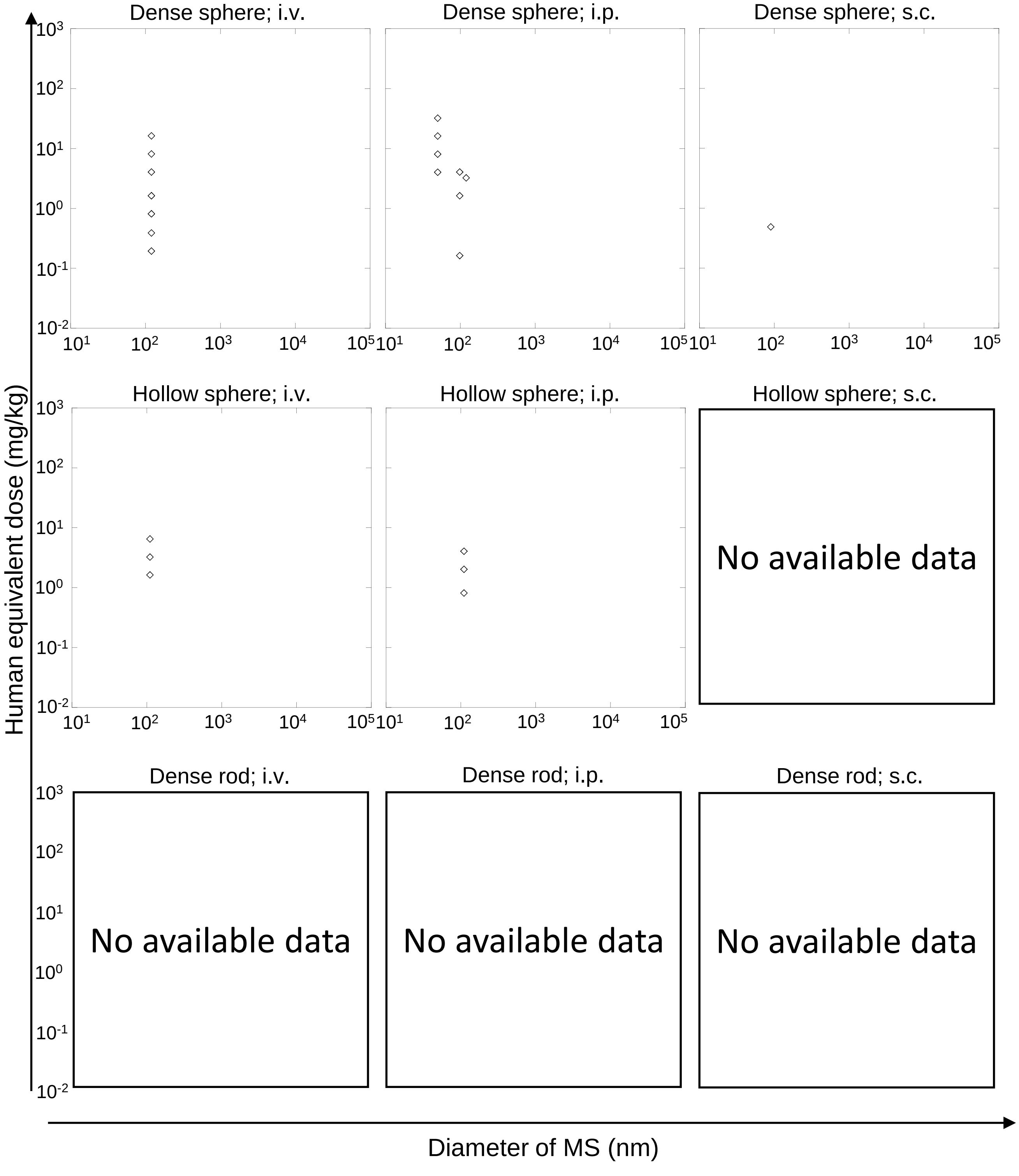


**Fig. S5 Repeated administration of MS particles with various shapes and internal structures**

Open symbol: no animal death; filled symbol: at least one animal was dead; rhombus: data obtained in the literature study; circle: obtained in the experimental study.
